# Dropsy

**Published:** 1826-04

**Authors:** 


1. *Researches into the Nature and Treatment of Dropsy in the Brain, Chest, Abdomen, Ovarium, and Skin; in which a more correct and consistent Pathology of these Diseases is attempted to be established, and a new and more successful Method of treating them recommended and explained.* By JOSEPH AYRE, M.D. Member of the College of Physicians, &c. 8vo. pp. 242. London, 1825. Longman and Co.
2. *A Statement of the Early Symptoms which lead to the Disease termed Water in the Brain; with Observations on the necessity of a Watchful Attention to them, and on the Fatal Consequences of their Neglect.* By G. D. YEATS, M.D. F.R.S. &c. Second Edition, considerably enlarged. 8vo. pp. 164. London, 1823. Phillips and Co.
3. *An Essay on the Nature, Causes, and Treatment of Water in the Brain.* By WILLIAM SHEARMAN, M.D. Member of the Royal College of Physicians, and Senior Physician to the Royal West London Infirmary. 8vo. pp. 123. London, 1825. Underwood and Co.

THE work of Dr. Ayre is placed first, since, with equatorial magnitude, it embraces the whole region of dropsies. We shall best discharge our duty by presenting a separate analysis of each production, and, in the execution of it, the reader will be pleased to recollect that we record, not our own opinions, but those of the respective authors.

Dr. Ayre acknowledges no single author to be his guide and authority, but confesses that he is indebted for many important facts to the writings of Wells, Blackall, Parry, Abercrombie, and Duncan. Fifty-one pages of his performance are dedicated to the pathology of dropsical affections. He thinks dropsy, whose essence is considered to consist in a serous effusion, to be only one, and not always the last, of a series of effects, which arise from a morbid condition of the serous and cellular tissues of the body. He denies that this effusion occurs from any want of tone in the absorbent and exhalant vessels, or from a mechanical obstruction to the return of the blood by the veins; and asserts that it originates from some particular action analogous to inflammation, or indeed inflammation itself, of various grades. This subject need not be pursued farther, as the pathology of each hydropic disease will be entered into under its respective head, separately and successively. The author offers observations on the presence or absence of serum in the urine of dropsical patients, and enriches his pages with a very long extract from an Essay* of the late Dr. Well's. We will also make a short transcript, since the facts of it are valuable and interesting, and must be highly acceptable to every class of readers.

" 'Among twenty-nine cases of dropsy of the skin,' observes this writer, 'which were not preceded by any disease to which dropsy is generally attributed, there were twenty-three with urine containing serum. The proportion of these cases, therefore, to the six without serum in the urine, being nearly as four are to one, is very much higher, than that of the whole number of instances of dropsy with serous urine to the whole number free from it, this being only as seventy-eight are to fifty-two, or as three to two. The proportion of considerable cases in this class, is also very great; for out of five instances of the urine being made firmly solid by heat, and of seven being made a soft solid by like means, which occurred among one hundred and thirty cases, two of the former, and four of the latter, were found in the twenty-nine cases, which form the subject of the present article. Of the remaining seventeen cases with urine containing serum, the quantity of serum was in nine considerable, though less than in those of the two preceding divisions; the urine of three had a still less quantity, and that of five but a small one.

" 'On the other hand, of nine cases of dropsy of the skin, apparently arising from weakness, the urine in seven was altogether without serum. Two of the latter occurred in old dysentery, one in chlorosis, one in chronic rheumatism, two after agues, and one after profuse bleeding, which had been employed to remove a great inflammation in the chest. —In the eighth case, which took place after ague, and in the ninth, which occurred in chlorosis of very long standing, there was a small quantity of serum in the urine.

" 'The presence, therefore, of serum in dropsical urine seems to be independent of weakness. It would appear, on the contrary, from the full and frequent pulse which frequently accompanies it, to be connected with too great action in some part of the system. This is rendered further probable, by the patient's often suffering great pains in the loins and

* Medico-chirurgical Transactions of 1812, vol iii.

limbs, both before and after the appearance of the dropsy, as was formerly remarked. An argument, indeed, may be brought against this opinion from the fact, that there was no serum in the urine in six of the cases, which had not been preceded by any other disease. But not to mention that weakness may arise without any previous *apparent* disease, those who are acquainted with hospitals will readily acknowledge, that it is often very difficult, and sometimes impossible, to obtain a tolerably accurate account of what has happened to patients, before their admission.

" ' In one case of dropsy of the skin, which followed quickly the application of cold to the body, and was attended with catarrh, and in another, in which the liver was evidently diseased, though there was no perceptible fluid in the abdomen, the urine contained no serum.

" ' The most common cause of dropsy of the skin in this country, or rather perhaps, the circumstance which most commonly precedes it, is a disease of the chest, manifested by cough and difficulty of breathing. I examined the urine in thirty-seven cases of this kind, to discover if it contained serum, and found it in twenty-four. In three of these the urine became a soft solid, upon being heated; in two, the quantity of serum was great, but less so than in the preceding three; in five, the quantity was still less; and in fourteen, it was very small. In the whole twenty-four there was probably extravasated fluid in the chest; but I have placed them under the title of dropsy of the skin, as there was commonly no symptom of considerable disease of the chest.'" 34.

The inflammation connected with dropsies may be sub-acute or chronic—symptomatic or idiopathic—local or general. The principal forms of dropsy are,—1. Hydrocephalus Internus—2. Hydrothorax—3. Ascites—4. Ovarian—5. Anasarca.

1. *Hydrocephalus Internus*. When effusion occurs in the brain, it may arise either as an incidental effect of acute inflammation of that organ, and of which the morbid result is coagulable lymph or pus; or of serous inflammation which is relatively in a sub-acute, or chronic state. The effusion may be either idiopathic or symptomatic. The former may be induced by a scrofulous diathesis, which favours the development of a local disease, and this may spring from direct excitement, or a general constitutional irritation of a specific kind. The latter, or symptomatic, may originate from structural disease within the head; or, secondly, from some relative weakness of its venous system; or, thirdly, from a distant irritation in the digestive organs. When the attack proceeds from the first of these causes, it is usually severe, and answers, in its symptoms, to the water-stroke of the German writers. When, from the second, the disease corresponds with serous apoplexy; and when, from the third cause, the turgescent state of the brain, which gives origin to symptoms that simulate those of the true affection, arises as a direct effect of chylopoietic disturbance, and is remediable, until arterial re-action commences, by means directed solely to its remote cause. The symptoms of hydrocephalus internus need not be narrated here, since they will be particularly related under the succeeding analysis. From the pathological principles explained, the same general modes of treatment will be applicable to all dropsical affections, subject only to certain obvious modifications. The following are the indications of cure, namely; 1st, To remove the visceral, or such other disease or state, whether local or general, which, when present, proves a remote cause of the effusion; *2dly*, To remove the morbidly increased action in the serous membrane, or tissue, which is its proximate cause; *3dly*, To promote the absorption of the effused fluid. The treatment of the disease in question will vary according to peculiar circumstances. If there be any causes acting through the system, agreeably to their precise nature, diuretics, diaphoretics, and aperients, conjoined with the occasional use of the warm bath and leeches to the temples, are to be employed, aided by such means as are serviceable in counteracting the tendency to the scrofulous diathesis. An issue is highly useful for an incipient turgescence, or impending inflammation of the brain. Should there be any structural disease within the head, a serous inflammation in the ventricles may be apprehended. Here, rigid attention to regimen is demanded, also cupping and leeching, and a seton in the neck, for the object is to avert, and not to remove, inflammation. Where the digestive organs are disturbed, and have caused a turgescent state of the brain, the primary seat of this disturbance is usually in the liver. By correcting and increasing the secretion of the bile, and disburdening the bowels, the sympathetic affection of the brain will disappear. Cupping-glasses, or leeches, are to be applied to the temples; a small dose of calomel nightly; and, in urgent cases, two or three times in the day, and a laxative enema, or an aperient draught on the succeeding mornings, assisted by diuretics, and exact regimen are indicated. Inflammation from structural disease is generally fatal, yet energetic antiphlogistic treatment is indispensable. When the inflammation is idiopathic a bleeding from the temporal artery is proper for patients above childhood; or, from the arm, with copious local depletion from the temples, is, perhaps, preferable. When leeches are used for infants, they should be three or four in number for an infant under six months; or five or six for one above a year in age. The bleeding from the leeches is to be encouraged, after their removal, by applying bags of flannel containing bran, and wrung through hot water. Twenty-five or thirty leeches may be recommended for adults; and, in either instance, it is desirable to induce a certain degree of faintness. Other remedies, already specified, are to be called into action. The treatment in every case should be persisted in, notwithstanding effusion appears to have occurred, for even here cures sometimes take place. To promote the absorption of the effused fluid, little, which is satisfactory, can be advised. The risk of renewing inflammation in the brain must be avoided; the head must be occasionally blistered; the diet spare; and the several secretions, especially those of the kidneys, ought to be cautiously promoted. The subject of hydrocephalus internus has been necessarily curtailed, because it will come twice under consideration, and at some length, under the analysis of Dr. Yeats's pamphlet, in this article. Effusion of water into the brain is a not unfrequent effect of blows on the head, or of scarlet and other fevers.

2. *Hydrothorax*. This may be either idiopathic or symptomatic, and proceed from a local or general cause. When idiopathic, it may either arise from all the local or general causes of inflammation, and, in this form exist, independently of, or in combination with, some higher grade of local vascular excitement; or, it may originate from a local congestive state of the vascular system within the chest, as an effect of general plethora; and, in those several cases, the effusion may prove more or less remedial of the local, and, sometimes, of the general excitement. When symptomatic, it commonly occurs under a chronic form, and from the chronic inflammatory action of the visceral disease extending to the serous membranes of the thorax. The occurrence of the effusion, when thus symptomatic, affords no standard by which to judge of the danger of the visceral disease; while the effused fluid, when in considerable quantity, is destructive to life by interfering with its necessary functions. The disease frequently has scarlatina as a remote cause of it, and is then usually preceded by slight œdema about the neck and chest, and is commonly accompanied by decided marks of vascular excitement. The urine, in these cases, is often of a brown hue, and loaded with serum; and the progress of the affection is sometimes so rapid as to destroy life on the second day. A certain plethoric state of the circulation, induced by intemperance and idleness, is a predisposing cause of hydrothorax, and one of important influence. In some individuals, past the middle of life, this plethora may produce sanguineous apoplexy by a rupture of vessels, or the serous one by arterial re-action, and consequent serous effusion. When inflammation assails the chest, it may, according to its degree, cause either pus, or coagulable lymph, a serous effusion, or a mixture of these products; but it is the serous effusion which is the characteristic of the disease under consideration. By the pressure of the fluid upon the vital organs contained within the thorax, a disturbance is given to their action, which is denoted by the following symptoms. A sense of oppression at the lower part of the sternum; an habitual difficulty of breathing; a cough which is either dry, or moist from a little mucus; an impediment to lie down in the recumbent posture, and likewise occasioning an impending suffocation, and an aggravation of the cough; sudden startings from sleep; an intermitting or irregular pulse; thirst; scanty urine, and œdema of the extremities. These symptoms, not singly but collectively, are pathognomonic of the effusion. They are, however, only symptoms of it, and not of the existing disease, for there are no signs which clearly point out that state. Hydrothorax and ascites resemble each other so closely in their remote and proximate causes as to allow their treatment to be considered together.

3. *Ascites.* Abdominal dropsy proceeds from nearly the same causes as the thoracic, and, like it, is most commonly symptomatic of some diseased viscus by the chronic inflammation in the internal tissues of the affected organ being propagated to its peritoneal covering, and thence through the cavity of the abdomen. The effusion is of inconsiderable importance compared with the visceral disease, which is its remote cause; until, from the risk of increased disease in consequence of distention, a necessity arises for tapping, when this operation proves a farther cause, perhaps, of aggravating the disease of the affected viscus, and either of renewing or of extending the hydropic excitement, or of converting it into a high or more destructive form of inflammation. Any diseased alvine viscus may occasion ascites, but most generally it is the liver, spleen, or mesenteric glands. Nor does the serous discharge always occur in every case in which these organs are morbidly affected, but only when their peritoneal covering participates in their disease; for the chronic inflammation extends from the cellular tissue of the internal structure of the organ to its investing serous tissue, and thence, as from a point, it spreads through the whole of the peritoneal investiture, with varying degrees of rapidity, and thus deposits an effusion, which is designated dropsy of the abdomen. Thus far have we advanced without indulging in a single extract, but here we will offer one, and only one, as a specimen of the author's style of writing, and mode of treating an interesting subject, and which may relieve the dryness of analytic detail.

"The morbid excitement, when once established in the peritoneal membrane, continues essentially connected with its primary cause; and the gradual increase of the disease within the organ, is followed by a correspondent increase in the intensity or extent of the serous inflammation in the peritoneal surface. The rapidity of the accumulation will be governed, therefore, by the intensity or extent of this excitement. Prior to the first tapping, in symptomatic cases of ascites, the accumulation of the water proceeds much more gradually than subsequently; for by the tapping, a cause of farther serous inflammation is generally superadded to the original one. After each successive discharge by tapping, therefore, the water is commonly renewed more quickly, and on one of these occasions, perhaps, a sub-acute or chronic inflammation is induced in some part of the peritoneum, by which a farther disease of that membrane is occasioned; and, at length, either by the increase of this superadded disease, or as an effect of some succeeding operation, a still higher degree of inflammation comes on, which may prove destructive of life.

"In some instances, where there is considerable disease of the liver, the water of the first accumulation may be absorbed and carried out of the body, and the patient may thus undergo a cure as it respects the effusion; but the serous inflammation, which caused the discharge, has only, in this case, yielded to an inflammation of a higher grade, which may arise, either, from the peritoneal membrane participating in the increase of disease in the affected viscus, or, by there accidentally supervening upon the secondary one a farther cause of inflammation. In these instances, as well as in those in which a higher inflammation succeeds the operation of tapping, coagulable lymph is poured out, by which the peritoneal surfaces, which were formerly the seat of the serous inflammation and effusion, are perhaps agglutinated together; and a fresh and more formidable disease is thus superinduced upon, or superadded to, the former one. In the worst of these cases pus is discharged from some points of the inflamed surface, and which, by mixing with the lymph and serum that are poured out at other parts, forms an apparently homogeneous fluid of a milky colour, which in puerperal and other cases of abdominal inflammation, has been strangely believed by some to be chylous; and by others, an effusion of the milk by a metastasis." 96.

The symptoms which attend the effusion of water into the peritoneal cavity are exceedingly obscure, and its presence can rarely be detected, until it is collected in such quantity as permits fluctuation. The remote cause may be either symptomatic or idiopathic, and either local or general. When the watery accumulation is considerable it may, *per se,* be a source of danger.

*Treatment of Hydrothorax and Ascites.* To subdue the chronic excitement of the serous membrane, and the primary chronic inflammation of the diseased organ, five or six ounces of blood are to be drawn from the integuments of the chest by cupping or leeching, and that side is to be selected on which the patient can most easily lie down, and the operation, to the amount of half the quantity, is to be repeated every third day, for three or four successive months, and, occasionally afterwards, as circumstances demand. A somewhat active blistering must be super-added, and conducted upon a similar principle. Where general plethora obtains venesection is admissible, but is seldom required, since it has no control over actions unmixed and chronic, whose cause and seat are local. Ultimately a seton may be fixed in the thoracic integuments. To employ mercury freely in every case of ascites is improper, and, beside this, under a salivation, the urine becomes charged with serum. Salivation then is to be shunned, but when mercury is exhibited, in minute doses, it may prove highly useful. Drastic purgatives are eminently beneficial, not merely by removing the water, but by subduing the cause of it. The best is gamboge; four or five grains for a single dose, combined with aromatic powder and super-tartrite of potash; and, in urgent cases of hydrothorax, ten or twelve grains, divided into four doses, and one administered every three hours. This medicine generally produces considerable watery evacuations from the bowels, and this is the specific object of its exhibition, and when fully attained, the oppression in respiration, and other distressing symptoms, whether of the thorax or abdomen, are much relieved. When the strength admits, the gamboge may be repeated once in four or five days, and, in general, it is borne better in ascites. "In ascites," observes Dr. Ayre, "I have seen *upwards of three gallons* discharged (by gamboge) in the course of the day from the bowels." But this practice is forbidden, when a disease of the liver or mesentery prevails as a remote cause, for here there is a tendency to diarrhœa, and, in the mesenteric affection such a proceeding would speedily destroy life. In addition to the means stated, diuretics follow, and are of vast importance, for they act on other emunctories as well as the kidneys. The most approved form is something less than a grain of squills, and only a sixth part of a grain of digitalis, made into a pill, and given uninterruptedly every third or fourth hour. To render them more diuretic, a third, or the half of a grain of calomel, nightly, is to be recommended, and followed by an infusion of dandelion, or any other popular diuretic decoction, as a common drink *ad libitum.* The preceding observations apply to the treatment of hydrothorax and ascites when arising from a serous inflammation of the investing membranes, in consequence of a chronic inflammation of one or more of the viscera. We are now to notice the treatment proper for the idiopathic forms of the hydropic inflammation, which may be either strictly local, or consist in a general specific excitement of the system, leading to a general watery effusion; and of which the exhalants only partake in common with the rest of the serous tissues. Here the pulse is hard, and venesection becomes an important remedy. The blood exhibits strongly the buffy coat, and the urine coagulates freely when subjected to heat. By a copious bleeding, the disease is sometimes at once arrested, and the water afterwards absorbed. The means, previously detailed, are as applicable to this form as the other, observing the cautions inculcated, and making due allowances for individual peculiarities. The operation of tapping will be considered under ovarian dropsy.

4. *Ovarian Dropsy.* This arises from a chronic inflammation, usually commencing first in the substance of the ovarium, and thence extending to its investing serous tissue. The premonitory symptoms are necessarily various, because any disease seated in that organ, whether scirrhus, or any other chronic kind, is capable, by an extension of its proper inflammation, to generate that excitement in its serous tissue which leads to watery effusion. Disease of the ovarium does not always occasion dropsy, yet, when it does, the progress is generally slow, and frequently stationary. The effused fluid, whether in small or large quantity, will often be retained in its sac for several years, and without much inconvenience or apparent injury to health. Next, it suddenly increases, and renders tapping necessary, and from this operation, a fresh cause of morbid excitement is afforded to the internal surface of the sac, and, perhaps, to the primary disease of the ovarium, and thus the fluid re-accumulates in a short period. Finally, each successive operation renews irritation and the operation itself, until a state of disease is induced within the sac, analogous to what occurs after abdominal tapping, which ultimately excites a degree of inflammation detrimental to existence. The remote causes of the different forms of ovarian disease are exceedingly obscure. External injuries may produce inflammation in this organ, and consequent scirrhus, or some other chronic disease; or the uterine irritation from the cessation of the catamenia may be favourable to its occurrence. Ovarian dropsy may arise at any age from puberty, but is most common in middle life.

*Treatment.* This form is never attended by the serous state of the urine, and requires a somewhat different management than the preceding species. Yet, after repeated tappings, the hydropic diathesis does prevail. In the early periods there is little debility from the effusion, nor any disturbance to the vital functions. The quantity of urine is only partially lessened, there is no thirst, and the general health is unimpaired. The disease is purely local, and demands occasional leeching and blistering, diuretics, a spare regimen, the warm bath, and moderately open bowels. The object is to subdue the chronic inflammation in the organ, which is the remote cause, together with the corresponding action in the investing tissue, which is the proximate cause of the effusion.

*Tapping.* This operation becomes necessary when the effused fluid is irremovable by medicine. It should not be performed too early, and in simple ascites not until all means have failed, while under a condition of visceral disease success is highly problematical. The best time is, when, from the large accumulation of water, difficult breathing, and other unpleasant and painful symptoms are occasioned. Where much visceral disease exists, tapping may cause a destructive inflammation in the peritoneum.* When a patient is unable to lie down in bed the operation is to be performed, for the fluid is acting as a mechanical irritant to the peritoneal membrane, and, independently of other obvious evils, suffocation may result. Prior to tapping, a few grains of gamboge may be given on the previous day, or some other drastic purgative; and six hours after this operation, a few leeches should be applied to the ab-

---

* To us this appears to be an unhappy explanation of a well known fact, for, in our judgment, a comparatively healthy peritoneum will be more likely to run into inflammation than a peritoneum long familiar with irritation induced by visceral disease. The truth is, and it rests on the highest authority, peritoneal inflammation usually occurs from the wound made in the peritoneum by the trocar not healing by the first intention, and permitting the water to dribble out constantly yet gradually. Thus is immediate irritation and consequent inflammation propagated from the open wound to the whole of the membrane, analogous to what takes place from punctured wounds into the thorax and abdomen. Sometimes the irritation alone exhausts life without the supervention of inflammation, and, of which, an instance happened in our own practice this year.—*Rev.*

domen; also a small blister on each side of it, and this ought to be repeated as early as the skin will permit. Again, the several remedies, previously used, must be re-exhibited, whether appropriated to the primary and secondary causes of the effusion, or the promotion of the absorption of it.

5. *Anasarca*. This disease consists of a serous inflammation in the cellular tissue of the body terminating in effusion. It may be either general or local, and spring from causes either idiopathic or symptomatic,* general or particular. Anasarca occurs under two forms, one being of greater intensity than the other, and it derives all its importance from the nature of the remote cause. When idiopathic, and proceeding from cold, it is usually, unimportant, for it readily subsides under proper treatment, and the effusion proves either partially or fully corrective of its cause. Thus anasarca serves the desirable end of obviating effusions into the brain, or other cavities, and is an event rather to be wished for, than dreaded, under such salutary circumstances. In some cases the effused fluid is of a highly viscid or gelatinous quality, so as to resist the pressure of the finger. This is a form commonly local, and often met with in the lower extremities of the aged, arising from cold locally applied, and sometimes occurring as a critical action on the declination of fevers. The uneasiness of it is considerable; and in its sensible appearances it closely resembles phlegmatia dolens, to which it is closely allied. Sometimes the inflammation yielding, the serous effusion of one part appears to be translated to another, and most generally to some other part of the cellular tissue, but occasionally to the serous membranes of the brain, or to those of the thoracic and abdominal cavities. Yet the most common form of anasarca is that which is symptomatic of visceral disease: and which, as it ordinarily appears, arises from an hydropic diathesis. It usually begins in the lower extremities, and is rarely accompanied with the strong marks of local excitement so obvious in the idiopathic kind.

In treating anasarca, we must advert to its nature and causes. If idiopathic, and unconnected with any other dropsy; if the pulse be soft, and the urine free from serum, the sole object is

* The writer entertains an opinion that anasarca, as well as hydrocephalus, may arise from some disturbance in the digestive function, by which this and other distant irritations are produced, through the operation of that obscure law of the animal economy, denominated sympathy. This, whether true or false, evidently resembles the sentiments of Dr. Yeats, and, throughout his work, Dr. Ayre treats that physician with a marked neglect, which his modesty, good sense, and merit do not deserve.—*Rev.*

the absorption of the effusion. Here recoveries need never be despaired of under any treatment, inasmuch as the absorbents will remove the fluid without any assistance; still, however, puncturing the œdematous parts, with the employment of frictions and of bandages, diuretics, and other means already stated, ought not to be disregarded: yet punctures and bandages are inadmissible until the cessation of the serous inflammation. To œdematous swellings, in which serous local inflammation, whether symptomatic or idiopathic, still subsists, leeches and cold evaporating lotions are advisable. Where anasarca originates from general excitability, denoted by the pulse and serous quality of the urine, venesection becomes necessary, aided by leeching and other means already specified. In cases seen late, and in which nothing dangerous is indicated, the promotion of the absorption of the effused fluid is alone needed. The state of the pulse, and of the urine, and the presence or absence of vascular excitement will be the safest guides of practice. When anasarca is considerable, long protracted, and symptomatic of visceral disease, with a serous state of the urine and a general failure of the strength, the cachectic state is established, and the treatment is beset with difficulties. General remedies once useful are now useless, and at times hurtful. The plan is to assist digestion and assimilation, and to correct the cause of the effusion by local depletion, blistering, and other rational means. The diet of anasarcous patients should be plain and unirritating, and, in the idiopathic state, rigidly antiphlogistic. The clothing ought to be moderately warm, and such as promotes insensible perspiration. Chamois or wash-leather drawers, and a similar waistcoat are to be worn next the skin, and are infinitely preferable to flannel or fleecy hosiery. The work, whose analysis is now concluded, contains cases and dissections, thirteen in number, illustrative of the different pathological conditions propounded in it, and are selected from the writings of Golis, Abercrombie, Cheyne, Home, and Blackall.

Our opinion is, that Dr. Ayre has produced a valuable work on dropsies, and one which may be read with pleasure and advantage. There is in it nothing which is either very striking or masterly, but it presents, in a compendious form, a general view of the pathological and therapeutical principles of the day with respect to dropsical diseases. The ideas scattered through various recent works of merit the author seems to have imbibed, to have brought them to the test of practice, and to have modified and enlarged them as his experience warranted, and his judgment sanctioned. This we conceive to be a fair statement of the nature of his production, and a just estimate of his pretensions as a writer. We differ from Dr. Ayre on some points, practical and doctrinal, yet on these we need not particularly expatiate. We think he is far too confident in the probability of curing hydropic affections; and, especially so in regard to ascitic and ovarian dropsies. It is freely admitted that ascites may be cured; still, however, an extensive observation of nearly thirty years, in private and hospital practice, has demonstrated to us that the cures of it are few in number. But the author goes farther, and says he has occasionally removed ovarian dropsies. Here we are compelled to be somewhat sceptical, and to question the powers of medicine; and, in this opinion the brightest names concur. The smallness of the ovarium in health, the insidious and occult nature of its morbid affections, the length of time which elapses, and the immense size it ultimately attains under dropsy, render all hopes of a cure by medicinal means exceedingly fallacious. But Dr. Ayre adduces a successful case, yet unfortunately it is narrated too loosely to establish conviction. The public is simply told "the fluctuation was quite distinct, and the body was enlarged to the extent of what it is in the fifth month of pregnancy." Now at this period of impregnation "the fundus of the uterus only extends about half way between the pubes and umbilicus."* Is this an indisputable specimen of ovarian dropsy? Is it in this superficial manner a case should be related, which has for its object the subversion of an opinion founded on the experience of ages? For any thing which is on record, the case might have been ascitic, or no dropsy at all, since nothing is more deceptive than incipient fluctuation, even where it does exist, and it is notorious that it is often simulated by flatus. It is true, that Dr. Ayre mentions another case of ovarian dropsy, which really seems to have been cured, but here, according to his own account, the cure was attributable to violence, and not the result of medicine, which is the point at issue. It is unphilosophical, whether in disputation or argument, to attempt to prove a rule by an exception. Nature sometimes delights in transgressing her own laws, yet, still they are laws, comparatively immutable ones, and a singular violation rather establishes, than subverts their permanency. One man perishes from a scratch on his leg;† another lives after the shaft of a chaise has been driven through his chest;‡ but still the former is an insignificant, and the latter a dangerous accident.

* Paris and Fonblanque on Med. Jurisp. Vol. i. p. 240.

† See Lectures on Surgery, &c. &c., *passim.*

‡ Case, by Mr. Maiden, 4to. London, 1812.

Although it would be wrong to affirm that Dr. Ayre is either a bad or incorrect writer, yet, he is rather deficient in the elegant simplicity and nice precision which characterize an excellent practical author. He occasionally employs words in a loose and ambiguous sense, and of this a single specimen shall be given, while, with respect to other parts of his style we will refrain from comment. "The effusion of the serous fluid is to be considered as arising from *a local vascular excitement in a serous tissue, which may be termed serous inflammation.*" Here, vascular excitement is assumed to be inflammation, which it is not. Is this really the author's opinion? or is it only attributable to looseness of phraseology? We are willing to credit the latter, since impropriety of language is more venial than erroneous pathology. Vascular excitement is not a state of disease, but one bordering on it, which may, and does indifferently terminate in sanity, or in morbidity. He that is elevated with champagne, suffers considerable vascular excitement in his brain, which may, and sometimes does end in inflammation or apoplexy, but much more commonly it ceases when the cause no longer operates. Inflammation is a positive state of disease;—true, it is preceded by, and accompanied with vascular excitement, yet, there is, likewise, an obstruction in the vessels of the inflamed part, attended with an increased action of the surrounding ones. This we believe to be the true pathology of inflammation, and although different theories prevail, it is presumed that none will deny a state of obstruction,* the essence of inflammation, and this is all we contend for, and which obstruction relieves itself either by resolution or adhesion, effusion, suppuration, or gangrene.

We think Dr. Ayre has erred, in exclusively attributing dropsies to either local inflammation, or the phlogistic diathesis; for though these may be the principal and general causes, we are persuaded, that hydropic affections can and do arise independently of them. What is a dropsy? An accumulation of serous fluid in a circumscribed cavity! Who can determine, that this invariably originates from inflammatory action? How often does dropsy occur in a cachectic habit, or in one debilitated by profuse hæmorrhage, and in which no suspicion of inflammation is credible, or the semblance of it discoverable after death? Other obvious causes need not be dwelt upon.

---

* "Almost every theory has been built upon the supposition, of there being some kind of obstruction in the inflamed part."—*Samuel Cooper on Inflammation.*

Indeed, the experiments of Dr. Seeds* clearly demonstrate "that large and excessive bleedings are *always* attended with effusion of fluid into the ventricles;" and these facts alone disprove the position under consideration. Dr. Ayre, also, assigns anasarca and œdema to inflammation, doubtless rightly in many cases, yet in numerous others without a shadow of probability. On this subject he observes, "the serous effusion of one part appears to be translated to another. The translation, however, in these cases, is not of the serous fluid, but only of the serous inflammation yielding the fluid."—p. 108. Here is a striking and accidental proof of that looseness of language to which we have adverted. In the first sentence an apparent fact is communicated, while in the second, it is denied, and an amendment substituted. Such sentences are like the witches in Macbeth,

> "That palter with us in a double sense;
> That keep the word of promise to our ear,
> And break it to our hope;"

since what is learned in the first, is to be unlearned in the second sentence, which is a most unhappy mode of gaining knowledge.

In our opinion the author would have written better had he laboured less, would have appeared to greater advantage had he discarded art for nature:—he seems to have been actuated by the laudable motive of obtaining fame, and we hope he will receive it; but he should recollect that the pace to eminence is slow and difficult; that design is easier than acquisition, that nothing great is ever hastily accomplished, and that the men who have acquired and deserved celebrity were remarkable for the simplicity of their style, exemplary for their long and patient observance of facts, and distinguished for the philosophical accuracy of their conclusions. Thus much have we said, because Dr. Ayre announces his intention of writing another book, and, therefore, we urge him to aim at obvious distinctions and not over-wrought refinements, to exercise a severity of judgment on his own opinions and success in practice, to adopt a compactness and precision of language, and then, we think, he will produce a work which criticism shall love to praise, since it will have little to censure.

The pamphlet of Dr. Yeats has been highly spoken of, and deserves to be analysed with considerable minuteness. It was

* Detailed at length in the Medico-Chirurgical Review, pp. 88 et 411. Old Series.

first published in the shape of a letter, but is now presented in a different and better form, and may be termed a medico-popular production, addressed to the profession, yet intelligible to the community. There are "some preliminary remarks" which may be read with advantage, and a "postscript of notes and observations," consisting of valuable extracts from the best writers of the age. These we must necessarily pass over, and confine ourselves to an analytic detail of what is still more important. The author observes,

"Since the first edition of this pamphlet was published, a great many cases have come under my care, which have established beyond all doubt in my own mind that the affections of the brain, especially in young people, which end in effusion there, if not guarded against, have their foundations in a great majority of instances in a deranged function of the powers of the epigastric viscera, and the many insulated cases, which have been published by several Gentlemen of the profession in the periodical journals, with a successful practical result founded upon the same doctrine, have given this opinion a currency upon philosophical and scientific principles. That complicated assemblage of symptoms which bewildered the practitioner and led him away like an ignis fatuus from the real nature of the disease, has been developed and analysed into greater simplicity, and the means of empirical experiment have become the means of rational practice.

"I intend these observations to apply strictly to that species of disease in young people, formerly so much involved in obscurity, which commences in a morbid action of the epigastric viscera, is in time communicated to the brain, and ends in a fatal effusion of fluid there; for although the ray of science has thus shed its light and dispersed some of those dark clouds which obscured the nature of this complaint, yet the precise condition and proper treatment of some of the diseases of the brain are still in part hidden from our view, for the symptoms of its morbid states are sometimes so contradictory, giving rise to indications of such contrariety, as to produce hesitation in the practical determinations of the most experienced; and this is sometimes caused by simple irritability of the brain, assuming all the appearances of a high inflammatory state there. I believe that in some instances of the former, bleeding has been injuriously performed, when in the latter it has been as injuriously omitted." v.

But, whatever importance Dr. Yeats may attach, and it is very considerable, to the idea that hydrencephalus (he uses this word and we shall adopt it during the analysis) in its origin, commences in organs distant from the brain, he is very far from denying that it, how often he cannot determine, does originate within the cranium. As we cannot afford space for every thing which is material, as the causes of this disease are involved in endless perplexity from contradictory opinions, as the author is very imprecise with respect to the meaning he attaches to vascular excitement and inflammation, and as his chief merit is a minute detail of the different trains of symptoms, preceding or constituting hydrencephalus, and of the appropriate modes of treating them, we shall best consult the reader's benefit by strictly devoting the attention to symptomatology and practice, from which something may be learned, undivested of hypothetical statements that often usurp the place of truth. This is a general observation, and disclaims any particular application; yet it is our duty to acknowledge that, as far as the epigastric viscera are concerned, Dr. Yeats is much disposed to hypothesis, and appears to be smitten with the rage of accounting for all morbid phenomena, without recollecting that the slightest exercise of ratiocination will plausibly answer every problem, but still the truth may be at an immeasurable distance. Assertion is not argument nor conjecture demonstration. Some readers think that he is singularly hypothetical with respect to the duodenum, and suppose he is the first writer who has attached great importance to it, yet here they have fallen into error. Dr. Hamilton* has written a section on the duodenum; so has Mr. John Bell;† while Hoffman has expressed opinions which deserve to be recorded on this page, and it is only just to state that the author himself has mentioned these authorities.—"Deficiente enim hoc motu peristaltico (duodeni) bilis quæ continuo effluit, ingenti in copiâ, accumulata et congesta in hac parte, mirificè intestinum distendit.‡"—"Quandoque etiam effectus pravorum istorum succorum, in duodeno primisque intestinis stagnantur, usque ad caput se exerunt et cephalalgias, vertiginem, torporem omnium sensuum, immò apoplecticos insultus ibi machinantur.§" —Dr. Monro|| has also discussed this subject, and given a description of the duodenum. Conceding, as we do most readily, every due importance to this intestine, we really cannot acquiesce in the premises and conclusions of Dr. Yeats, nor can we, with any propriety, consider the duodenum, as he does, to be a second stomach, presuming this term to be purposely employed to magnify its consequence. The stomach is the receptacle for food, solid and fluid; and its office is to convert this food into a chymous mass. This must be done, however large

---

* On the use and abuse of Mercury, Sect. vii.

† Anatomy, vol. iii. p. 278.

‡ Oper. Omn. vol. vi. p. 191, De Duodeno, multorum malorum causâ.

§ Idem. vol. vi. p. 192.

|| Edinburgh Medical Essays, vol iv. p. 57.

the quantity; hence it happens that such inordinate action of the organ occasionally generates morbid feelings, or disease itself. But how differently and favourably is the duodenum circumstanced? Digestion is a slow process, usually requiring three or four hours, and as soon as a portion of food is reduced to a homogeneous consistence, it instantly passes into the duodenum, without waiting until the remainder undergoes the same change.* While then, the stomach, in a few minutes, receives an abundance of alimentary matters, and has these to digest, and their noxious qualities to resist, the duodenum, most leisurely indeed, and during a space of three or four hours, has only to accept these matters piece-meal, not in their original objectionable state, but in a bland form, designated chyme, and which is slowly mutated and improved by the admixture of the biliary and pancreatic secretions. Where, we beg to know, is the analogy between the stomach and duodenum in regard to their functions?

According to Dr. Yeats there are three distinct trains of symptoms connected with water in the brain, and each of these he illustrates with narratives of cases, which he conceives to be appropriate. The cases come not within our scope, but the symptoms, and treatment of them shall be stated with scrupulous fidelity.

*First Train of Symptoms.* Occasional languor, as if from fatigue, with intervals of considerable activity. It is attributed to this cause, for the child reclines on the sofa, chair, or lap of its mother; and the usual healthy appearance diminishes, though not permanently, in a transient paleness and occasional collapse of the features. There is a dark coloured line under each eye, with a dulness of that organ; the softness and pliability of the skin lessen along with a consequent harshness and increased heat of the surface. The appetite is capricious, with irregular thirst, and a tardy state of the bowels; the tongue is white and disposed to aridity in the mornings. Sometimes a cough attends, which is often very teasing. The pulse exhibits no morbid change. The urine is at times higher coloured than usual. When, because the child is somewhat constipated, a domestic purgative is given, an attentive examination may discover an incipient diseased secretion from those glands connected with the intestinal canal, although the evacuation, more than commonly consistent and firm, presents no very striking

* Dr. Prout, in Thomson's Annals of Philosophy, 1819; and Wilson's Experimental Inquiry, &c. 1817.

alteration of colour. Occasionally the evacuation will be much lighter; at other periods only partially light; and now and then the whole appears tinged with a dark colour of a greenish cast, and is accompanied by more slimy matter than a purgative will produce. If the patient complain of his head, it is not of pain, either acute or dull, but of a disagreeable noise and confusion, with some giddiness, and a painful sensibility of the eyes. The scalp, at intervals, feels sore at friction or touch; and sometimes the neck is stiff. Now a puffiness will be felt, and also a fulness over the centre of the stomach, which extends to the navel. Pressure there causes uneasiness; yet these symptoms, like the others, are not lasting. The only symptom, observing any permanency, is the torpidity of the bowels, and this, though mutable, is always more or less present. The sleep is frequently disturbed by restlessness, and repeated movements in bed. Still the child is said to be only not well from improper food. We cannot, *à priori*, positively determine what precise disease will result from this condition of the system; but this irregular excitement, this vacillating state very generally leads to the next chain of more manifest morbid actions, which terminate in water of the brain. Hence especial caution is needful. It happens, occasionally, that the excitement of the cerebral vessels is attended with very severe ear-ache, and as this, beyond the pain of it, is usually unimportant, attention should be directed to the greater evil, and no warmth must be applied to the ear, which is injurious. When the symptoms already described are a prelude to hydrencephalus the children are often gifted with a precision of ideas and quickness of apprehension far beyond their years. Let then the practitioner be on the alert, let him not dismiss his little patient with an opinion that the symptoms are only referrible to an insignificant stomachic affection, for, if he do, he will again be summoned to behold the next more prominent symptoms, and to lament the errors of his hasty conclusion. The duration of this previous state, prior to its assuming an arresting shape, will depend upon the accustomed management of the child, its customs as to diet, air, and exercise, and its constitutional disposition, whether independent of, or connected with, these customs. Thus it is, the disease runs a rapid, or a protracted course. In some children, from whose constitutions the early impression of morbid action is easily removed, the occasional exhibition of a domestic purgative will be completely successful, but in the majority of cases such a practice is unsafe, and it is indispensable to combine an alterative with the cathartic, so that the accumulated diseased load may not be removed from the intestines, at once, by several large evacuations. Here a case is related, by the author, to prove the very sudden manner in which the disease surprises a child in apparent good health, "and hurries it into imminent peril before the parent is aware of any danger whatsoever." Recovery was the result of vigorous treatment, consisting of nine leeches applied to the temples, a dose of calomel, an opening draught, an active injection, and, finally, the pulv. scamm. comp. et hydr. submur. with a scruple of potass and sulph. in solution. The head and shoulders were supported in nearly the erect position in bed, and they were kept uncovered and cool.

*Treatment.* In chronic diseases there should be a gradual restoration of healthy action to the intestinal glands and to the alimentary canal in preference to many evacuations produced by a strong purgative. To change the kind of it is desirable, in order that the different glands, as well as the various portions of the intestinal tube, may be excited by varied stimuli. The quantity and quality of the foul discharges do not depend solely upon stagnation in the torpid bowels, for, morbid secretions are going on, and their mere dislodgement will not restore tone and health. An alterative effect is wanted to stimulate the glands to their healthy functions;—this done, in addition to the removal of the accumulated load, salutary discharges will regularly take place. A combination of the evacuants required, will occur to every practitioner, with proper intervals of exhibition agreeably to age and the degree of constipation. There are none better than extr. colocynth. comp. with calomel, or the pil. hydr. or the latter with aloes, rhubarb, or scammony, twice or thrice, or even four times, in twenty-four hours, in moderate doses, so as to excite the abdominal viscera gradually. In this state no general constitutional effect from mercury is called for, and to obviate such an effect and to evacuate the bowels and glandular system, a full purgative ought to be occasionally given, composed of senna and neutral salt, and, on that day, the mercurial medicines may be omitted. The puffiness and fulness about the region of the stomach are to be referred, probably, to the duodenum, whose important offices are too much overlooked. Calomel administered alone often does harm, and should always be conjoined with other cathartics.

*Second Train of Symptoms.* When the circumstances already detailed are disregarded or misunderstood, the symptoms assume a formidable and commanding shape. The occasional languor resembles permanent lassitude; the returns of activity diminish; an almost constant recumbent posture is wished for; the unhealthy look of the countenance becomes more lasting and observable; the darkness under the eyes is of a deeper colour; the febrile excitement is more regular and apparent; and there is a consequent harshness of the skin, with occasional flushes across the cheeks, and particularly on one cheek. The hearing turns more acute, the child starts at slight noises; transient pains are felt in the head, more or less acute and frequent, and, at times, when the child is comparatively comfortable, it will exclaim, "oh, my head aches!" Some complain of the head feeling sore to the touch. Now the pulse is occasionally much quickened, and, it will change its frequency on the least movement; though not particularly irregular, yet, if carefully examined under the febrile accession, an irregularity will be readily discovered, once, twice, and sometimes more in the minute. Periods of drowsiness supervene; the bowels are more obstinately torpid. When stools are procured, they are of a very disagreeable smell, and of a very morbid appearance; now and then a glutinous mass is intermixed with dark lumps of fæces, or there is a mixture of a deep green with matters similar to yeasty fermentation. The colour and appearance of the evacuations will vary much in the same person at different periods. Nausea, sickness, and vomiting are frequently troublesome, either when the little sufferer raises its head from the pillow to which lassitude and drowsines had consigned it, or after taking food. In some, the puffiness and fulness about the stomachic region are not now visible, since one part of the morbid actions has yielded to others of a more violent nature. This symptom, though common, does not invariably attend. All the symptoms shew evident marks of irregular excitement. A giddiness, with an unpleasant cloudiness in the sight, is complained of, and prismatic colours are occasionally seen; and, although the eyes display nothing morbid, yet, at times, they are blood-shot, and a strong light is disagreeable and painful. The urine varies much in colour and quantity, depending entirely on the febrile accessions. The appetite becomes deficient, but is, at intervals, morbidly keen. The thirst is troublesome; the tongue white and inclining to aridity. Even now the complaint is manageable; in some easily so, from previous customs antecedently dwelt upon; yet, be it recollected, every hour, at this juncture, is inexpressibly precious. Let it not be thought this assemblage of symptoms arises from foul bowels, for if their ultimate consequences in producing cerebral disease be disregarded, mischief will, as it has often done, result; particularly in making too nice a discrimination between the symptoms generative of water in the brain, and the infantile remitting fever, on which Dr. Butter has written so ably. At such a period a worm is passed, and this confirms an erroneous opinion, a fatal delusion, and an inefficacious practice.

*Treatment.* The plan must vary from that of the first train of symptoms, and be more active in proportion to existing circumstances. The means specified must be changed according to the form which the disease assumes. The feel and condition of the pulse, the increased febrile accessions and pain, evidently denote that an inflammatory tension has taken place in the circulatory system, requiring the loss of blood; but, whether locally, or generally, or both, must depend upon age and constitution, urgent symptoms, and medical judgment. Dr. Yeats has directed both in the same patient with decided advantage; most commonly detraction of blood is indispensable; most frequently the local is demanded. We need not be timid; that the disease springs from debility is a false principle; yet still large general bleedings are objectionable for they may contribute to the much-dreaded effusion into the ventricles. The author has never directed bleeding from the jugular vein, because venesection in the arm, and the application of leeches, have answered his purpose very well. General, must not supersede local bleeding, since the morbid condition of the extreme vessels, yielding the effusion, is independent of the action of the heart, and the strong action of that organ may be reduced without overcoming the specific state in the minute branches constituting inflammation there, and thus the re-action of local irritation will exhaust life.* Hence topical bleeding is so useful, aided by the powerful alterative effects of mercury. This inflammatory tension must not continue, for it will hasten

---

* This sentence inculcates an important, and ever to be remembered, practical truth. In some morbid conditions, general, must not supersede the local bleedings, for the practitioner "may bleed generally till the heart is killed," and yet the local morbidity will remain unsubdued. This fact is confirmed by the experimental truths of Philip, Parry, and Seeds. It clearly appears from the able experiments of Dr. Seeds, narrated at length in pages 88 and 411 of this Journal, that large and excessive bleedings are always attended with effusion of fluid into the ventricles of the brain. Thus, immoderate bleedings are occasionally hurtful in apoplexies, and other diseases. For farther information on this interesting subject, see Yeats's Letter to the Editor of the Medico-chirurgical Review, vol. i. p. 377, with the Reviewer's Reply, p. 470; also Kirkland on Apoplectic and Paralytic Attacks, p. 70, 71, a work containing many practical facts; and Dr. Crampton's case which is recorded in the first volume, page 176, of the Transactions of the Dublin College of Physicians.—*Rev.*

and augment effusion; and, as there is preternatural vascular irritation in the organs subservient to digestion, the application of leeches to the regions of the stomach and liver, as well as to the head, will prove essentially useful. For the same reason, the neutral salts, particularly the sulphate of potash, exhibited twice or thrice in the day, either in the infusion of roses or the saline draught, are always preferable at this time to the resinous purgatives, because more cooling. Two or three grains of calomel ought to be administered likewise every night without fail. In all inflammatory complaints a combined exhibition of calomel and the neutral salts, in small and divided doses, is a most powerful antiphlogistic; and that increased local vascular action occurs in various parts of the alvine viscera in this complaint, is indisputable, and is demonstrated, not only by symptoms, but by dissections. To apply blisters to the head is a nice point for discrimination; much caution is necessary; they may do harm there; at all events, they must be preceded by full evacuations. The diet must correspond with the treatment, and not be spicy, or stimulating, or consist of animal food, unless in the form of broth occasionally. At the approach of convalescence it will be generally necessary to give a tonic twice a day for a short period, and it is advantageous to join an aperient with it to induce and perpetuate a daily regular intestinal evacuation. A few grains of columbo, with a grain or two of rhubarb, twice diurnally; or, if the increased action have run high, or any disagreeable heat remain, an infusion of columbo, or of quassia, with a neutral salt (at this time the Epsom is best) will be the preferable composition. Here the writer records a case, "in which the lungs partook largely of the irritation previous to the affection of the brain."

*Third Train of Symptoms.* The accession of this state is marked with greatly increased violence, and with great suffering. The cuticular heat becomes intense and harsh; the febrile attacks strong and distressing; the pains of the head more acute, and more frequent in their return, and the loud screams of the child on this account are truly afflicting. The pupils of the eyes shew much dilatation, but still contract on the approach of light, though not healthily, by a waving languid vibratory motion. Squinting takes place at times, double vision is complained of, and when the patient is desired, though not then seeing double, to view an object "he sees the object not where it really is, but on one side of it, by pointing to the spot." There is a knitting of the eye-brows, with a distressful countenance. For a few minutes there will be a perfect silence and quietism, with a fixed steady stare of the eyes, and a very great dilatation of the pupils, when a sudden start will take place, with a loud screaming, and a quick tossing of the arms over the head. Frequent moaning; deep sighing: sickness and vomiting exist. The bowels are most obstinately costive; the evacuations, when procured, are very scanty and ill-formed, and extremely offensive; and when, by active means, a large mass is ejected, it is unlike fæces, being dark, yeasty, gelatinous, and smelling like a mixture of sour grains with putrid matter. The tongue is foul, sometimes brown and dry. There is much thirst; no appetite. The urine is irregularly secreted, both in colour and quantity. The pulse is very irregular in the tone of the vibration, as well as in the flow of the blood; sometimes slow, sometimes quick and intermitting with a tensive feel, until at last it sinks into permanent sluggishness, which ushers in its ultimate and fatal celerity. A dewy moisture settles in drops upon the upper lip and around the nose. A considerable wasting of the flesh has now occurred;—the countenance being pallid and sunk, with accessions of transient crimson flushes playing about the cheeks; a hollowness of the temples; blueness of the lips, and a frequent retraction of them from an attempt, but inability, to cry, ending in a whining tone from weakness. The eye-lids are half open and motionless; the eyes filmy and fixed with a peculiar stare from the extreme dilatation of the pupils. The circulation is extremely hurried; convulsions frequently take place; palsy supervenes, either partially or generally, and, if the former, the child is constantly sawing backwards and forwards across the body with the arm of the opposite side, until death, most commonly in one convulsive struggle, ensues.

*Treatment.* At the commencement, much will depend upon nice judgment, prompt discretion, and vigilant attention, since, however deplorable the condition of the sufferer may be, it is not to be considered as completely hopeless. We must diminish the vascular excitement* in the brain by repeated applications of leeches to the temples, by general bleeding if the force of the heart will bear it, by cold applications to the head,

---

* It deserves to be mentioned that Dr. Yeats, in various parts of his Pamphlet, sometimes writes of vascular excitement, and sometimes of inflammatory tension, leaving his readers in the dark, whether he uses them as synonymous terms, or, if otherwise, in what the difference consists. They are most certainly two opposite states, as we have explained, in a preceding analysis.—*Rev.*

and by stimulant applications to the legs. Our author once thought that recovery was impracticable after the occurrence of effusion, but subsequent experience has convinced him he was mistaken, and that, when death speedily ensues, it is assignable to the morbid excitement having destroyed the energy of the brain. Not unfrequently the patient shall recover from the dangerous excitement of this organ, with effusion of fluid into the ventricles, but without any paralytic affection of the limbs; yet there will remain a total loss, or a partial abolition, of some one or more of the senses, and most commonly of vision. The smell has been lost, and the speech left imperfect, or even destroyed. Sometimes with, or without, the consequent palsy, a morbid state of the brain will remain, occasioning severe head-aches, with spasms and convulsions occurring at intervals, and, at last, fatally terminating in a violent accession of disease in the brain. During this period, the child will grow with increase of flesh and strength, and with such an appearance of health as to induce friends to be credulous with respect to danger, and to discredit the melancholy prognosis which judgment expresses. One of the most common morbid consequences of apoplexy, is a great imperfection or loss of speech, and seldom or ever a loss of sight; while the latter is the most common in children who have, comparatively, recovered from the disease under consideration. Effusion is not an absolutely fatal event; but, the chances of cure will depend, perhaps altogether, upon the arresting of the excited state of the vessels of the brain, and, before the effects of depletion have become doubtful and dangerous from the debility which ensues, and also before much effusion has taken place. Together with the judiciously repeated application of bleeding and blistering, a diligent use, both internally and externally, must be made of mercury, which, when the constitution is influenced by it, powerfully assists in altering that morbid excitement constituting the disease. These, and daily evacuating the bowels, are means sometimes successful; for the effused fluids have been carried off by them, especially, under a general mercurial action. Still, it must be observed, that patients have died after the mercury had well affected the mouth, and recovered when a very large quantity of that medicine had failed to effect that purpose. No doubt, however, exists but that the habit may be influenced by mercury without the participation of the gums and salivary glands. Bleeding much disposes the constitution to the easy operation of mercury, whilst a high action in the system exercises an opposite effect. This is a valuable practical fact, and the public attention was pointedly directed to it many years ago by Dr. James Johnson. Diuretics are of little service until the reduction of the cerebral vascular excitement:—death sometimes occurs with much flow of urine, yet they are, nevertheless, useful from counter-irritation, by increasing the secretion of other glands. The digitalis deserves consideration for the other peculiar effects of this valuable and extraordinary medicine. The whole of the preceding observations are chiefly applicable to children from two to fifteen years of age, since they are most liable, although the disease may arise in those much younger, or even at mature periods of life. There are, indeed, two periods in the growth of children which are exceedingly apt to generate that state of the brain, terminating in an effusion of fluid, and these are dentition, and the efforts of the constitution to produce the catamenia. The frequent use of calomel as a purgative during teething is objectionable; and, during this irritation, considerable morbid changes in the alvine evacuations are apparent. Dentition frequently occasions great inflammation of the lungs. It deserves to be noticed that troublesome coughs are often removed by attention to the condition of the chylopoietic secretions.

Children are prone to hydrencephalus,* and much less so to general dropsy, while the reverse obtains with respect to adults. The former disease is not usually connected with the dropsical diathesis. The fluid in health, found in the ventricles of the brain, does not contain coagulable matter by the test of acids; yet, the hydrencephalic deposition displays, but certainly not always, a coagulable precipitation by heat or other tests.

The profession has already expressed a decidedly favourable opinion of Dr. Yeats's book, and, to us, there only remains the pleasing duty of echoing the public voice. Without subscribing to the author's peculiar doctrines relative to the chylopoietic viscera, or sanctioning the sometimes mechanical way in

---

* The best authors agree in attributing the origin of this disease either to disorder of the digestive organs, causes within the cranium, scarlet and other fevers, insolation, or blows on the head, but, so far as we know, no writer has related a case arising from a heavy load on that part. Such an instance occurred in our practice thirteen years ago. A young and healthy man, carrying a large box on his head, sank under it into a comatose state, but without being otherwise injured by the load. He lay in that state for several days; yet he could be roused from it, and knew our names. There were no convulsions, and little appearance of paralysis; he appeared like one in a deep sleep, answered questions, and instantly relapsed into the same soporific condition. He expired about the twelfth day, and, upon examination, there were general marks of inflammation, and a most enormous quantity of limpid fluid within the lateral ventricles of the brain.—*Rev.*

which he strives to elucidate certain morbid conditions, we will frankly bestow approbation on the work, and candidly acknowledge that it has done, and will do, much good, since it explains with talent, and treats with judgment, a train of symptoms incidental to children, which, whether they do, or do not, generally lead to water in the brain, are dangerous in themselves, and may become still more dangerous by neglect or mismanagement. Having commented on the style of Dr. Ayre, we should despise ourselves, and would be justly despised by others, if we refrained to observe that his language, compared with the conversational redundancy of that of Dr. Yeats, is conciseness itself. We hesitate not to declare that any man, moderately skilled in composition, may compress seventy of this writer's pages into fifty with advantage to the sense, and elegance to the diction.

The preface of Dr. Shearman, amounting to twenty-three pages, consists of various observations on the superiority of clinical practice over anatomical pathology, and in the propriety of which we cannot but concur. This subject, indeed, has already been discussed at some length in a former Number,* and, it is one highly deserving of serious attention. The causes of disease are frequently enveloped with mystery; their effects are usually ascertained too late; while the symptoms are obvious to the senses, and explicable by the understanding, and their immediate controlment, and final subjugation can be generally accomplished by acute observation, and sound judgment. It would be easy to express the peculiar opinions of Dr. Shearman in a line or two, but we conceive it will be more decorous to him, and more interesting to our readers to unfold them in regular analytic order.

Hydrencephalus signifies an effusion of watery fluid within the brain;—hydrocephalus, a similar effusion within the head; and both terms commonly designate symptoms prior, and subsequent, to this effusion.

"I am inclined to think that hydrencephalus, or water in the brain, is an accidental circumstance, occurring during the progress of several diseases, and is produced by a variety of causes; and that its occurrence in this case depends on the predisposition or previous state of the serous membranes of the brain in the individual; and that therefore the essential character of hydrencephalus, when the term is employed to designate any protracted series of symptoms, consists in that previous state of membranes, or predisposition.

"When we examine any history of the reputed disease, as given by

* No. 4, New Series. Buchan on Symptomatology.

authors, taking, for instance, that by Dr. Golis,* acknowledged to be one of the best, we find an assemblage of symptoms enumerated, common to a very great variety of diseases, but can fix upon none which are certainly diagnostic of hydrocephalus, as a distinct disease, until the arrival of that stage which indicates actual effusion, the accidental effect of the operating causes, determined by the predisposition or peculiar state of the serous membranes.

"That the effusion is accidental, determined by the predisposition, not an essential result of the existing symptoms, is evident, because these symptoms frequently continue for a long period, and sometimes terminate fatally, without producing effusion, in those children who are not predisposed thereto by the peculiar state of the serous membranes, viz. too great irritability and too augmented circulation." 9.

The preceding extract is rather too long, but it was unavoidable from an anxiety to do the writer full justice, since it is an epitome of the sentiments which he entertains. We now proceed with the analysis. The worm or remittent fever of children has a regular progress and termination, yet, effusion into the brain does not occur, unless in those possessing a state of predisposition; and if so, it is a frequent consequence. In the latter case, the whole train of symptoms, including the fever, is usually called *hydrocephalus;* whereas, in reality, the fever was not any essential part of the hydrocephalic affection, but only an exciting cause. The same reasoning is applicable to various other diseases, in which water in the brain takes place or not, according to the state of predisposition. Children of five or six years of age are frequently attacked with symptoms of fever, all of which are removed by antimonial medicines and mercurial purgatives; yet, if these means be neglected, an effusion into the ventricles is the result. Are these symptoms, then, at the first, to be termed incipient hydrocephalus, a peculiar, distinct disease of the brain, of which all the rest are symptomatic? or is not rather the effusion to be considered, not as a distinct idiopathic disease, but the simple consequence of that general vascular excitement which exists in the membranes of the ventricles in common with all other parts? The various senses in which the term hydrocephalus is used, satisfactorily explains the success or failure experienced by different practitioners. The symptoms denoting this disease have often been particularly noticed, yet, after death, no effusion has been discoverable. Cheyne, Quin, Warren, and Golis, mention such cases. Now, it is evident that, in these cases, the symptoms could, at no period of the disease, be produced by effused fluid;

* Gooch's Translation, p. 38, *et seq.*

and we cannot, therefore, with strict philosophical deduction, conclude, that in other cases, where effusion has been ultimately discovered, the symptoms, precisely similar in appearance and progress, were owing to the effusion. There are, indeed, strong grounds for supposing, that the effusion was the accidental, not essential, termination of the disease, which, in all the cases, was probably *fever* of some description, affecting the brain in a greater degree than other parts:—a usual circumstance in fevers, and, among others, in typhus.

Cheyne and Yeats* exhibit in their writings, decisive evidence of hydrocephalus being an accidental effect occurring in a great variety of diseases, when the former admits that "every different stage, certainly every different form of the disease, requires a considerable difference of treatment;"† and the latter physician acknowledges that "the watery effusions which take place after scarlet fever are produced in an analogous way to the effusion in hydrocephalus." Whether it be scarlet fever, or any other febrile affection, if there exist great vascular excitement in the brain, and great irritability in that organ, effusion may arise as an accidental occurrence, since it is not the result of a particular specific disease distinct from general fever. Indeed, the opponents of a contrary doctrine are compelled to assign the final event (effusion) as the only diagnostic; and, when this event has not occurred, they term the disease one *resembling* hydrocephalus; nay, when they have assured themselves of the existing disease being properly and specifically hydrocephalus acutus, dissection has demonstrated the brain to be perfectly healthy. The infantile remittent, the worm fever of children, and the attack of acute hydrocephalus are undistinguishable until decisive symptoms of the pressure of effused fluid are present. The event of such is the only diagnostic guide:—if recovery take place it was not, if death occur it was, hydrocephalus!—Again, effusion is frequently discoverable in the ventricles, without the manifestation of any febrile symptoms; and hence we cannot justly consider effusion into the brain as the proximate cause of these symptoms in any one case.

Viewing, then, dropsy of the brain as a mere effect of a certain state of the serous membranes of that organ, and this state being in some instances produced during the progress of disease, and in others arising from various causes, it becomes important to ascertain when this state exists, and by what symptoms it is discoverable. Two conditions of the brain may be considered

---

* See Early Symptoms of Water in the Brain, p. 124.
† See an Essay on Hydrocephalus Acutus, by J. Cheyne, M.D.

as more particularly predisposing to effusion during the advancement of acute disorders in children, namely, a greater rapidity of circulation in the vessels of the brain, and an excessive degree of irritability of that organ. These states may prevail, either singly or jointly; the danger of effusion being greater in the latter: they do not, *per se*, constitute disease; they may exist for a long period without impairing or interrupting any of the functions of life, and are, indeed, compatible with their most perfect performance. Such conditions of the brain are observed in children of active dispositions, and this increased state of the circulation must necessarily be productive of a greater tendency to exhalation into its serous cavities. When, then, febrile diseases occur in children, it is obvious that by still farther increasing the circulation within the brain, they may conduce to accumulation of fluid with great facility. Inflammation has been said, indeed, to be the proximate cause of hydrocephalus; but effusion into the ventricles is not preceded by inflammation; it springs from vascular excitement, and "something more is requisite to constitute inflammation than merely increased circulation."

"While the blood continues to flow freely through a part, no inflammation is produced, whatever be the quantity transmitted to it; it is when resistance is offered to the transmission through some of the vessels, that inflammation follows: the balance between the quantity sent to the part, and the disposition in the vessels to receive that quantity, being deranged, a state of disease commences; the resistance to the flow of blood acts as a stimulus to the vessels conveying that blood, and incites them to increased action, which still further augments the quantity of blood in the part, whilst, the resisting force not yielding to the impulse, symptoms are aggravated, and the mischief proportionally increases. Not only is the resistance not diminished by the additional impulse of blood, but it is actually augmented; the endeavour to distend the resisting vessels increasing their state of contraction, whereby their further distension is prevented. Should this struggle long continue, and the removal of such obstructing cause not be accomplished, other effects are produced, which constitute the different stages and terminations of inflammation, viz. effusion of coagulable lymph, adhesion, or suppuration." 41.

The above extract proves that Dr. Shearman makes a very judicious distinction between vascular excitement and inflammation, and that his sentiments on these interesting subjects do not materially differ from our own. He is, also, of opinion that increased and strong action of the vessels will, independent of actual inflammation, occasion the transmission of lymph, containing more coagulable matter than ordinarily circulates through them, and hence arises a certain appearance of opacity in membranes which are naturally transparent, e. g. the *septum lucidum* in some cases of effusion into the ventricles. To proceed with the analysis. The effusion in genuine hydrocephalus, appears to consist of merely aqueous fluid, neither coagulable, nor containing either albumen or fibrin. The character of the fluid, therefore, distinguishes it from the product of inflammation, as the effusion resulting from that affection always contains coagulable matter.* In some cases, however, described by writers on this malady, the effused fluid has been found coagulable, and of various degrees of turbidness. This occurs principally in that form denominated by Golis "the tumultuous;" yet, even this is only the consequence of strong vascular excitement, for there is no decisive evidence of inflammation; and the fatal termination may be justly attributed to the rapidity of the effusion, which alters the condition of the brain, and renders it incapable of performing its necessary functions.

The premonitory symptoms which, in children, denote undue irritability of the brain, or increased circulation through its vessels, and indicate, without the presence of actual disease, the existence of that state of the serous membranes wherein effusion is produced with great facility, are, great wakefulness, considerable sensibility to slight impressions, with restlessness, and animation in a high degree;—the retina is particularly sensible to light, the pupil is sometimes much contracted; and the child becomes fretful, and often cries without any apparent cause. In such a state, slight causes of disease readily excite disturbance of the cerebral functions; and, whenever fever arises from worms, dentition, constipation, or any other cause, the brain participates in a disproportionate degree, and yields effusion; while, under a different state, none whatever would occur. When effusion takes place, the danger is to be estimated rather by the rapidity than quantity of it; since, if slowly, the brain may accommodate itself to the pressure; but, if most rapid and large, as in the water-stroke of Golis, the functions of that organ are at once speedily overwhelmed, and death follows. Accordingly, the water-stroke is invariably fatal;—the acute hydrocephalus may occasionally be cured; or, in other language, the general disease of the system may

* In peritoneal and ovarian dropsy, arising from membranous inflammation, the fluid is not only easily coagulable, but portions of it are frequently found actually coagulated, so as to impede its passage through the canula in paracentesis, and long stringy shreds may be drawn out by the fingers. On dissection, in such cases, decided marks of inflammation are usually discovered, namely, altered structure, induration and accretion of membranes by means of effused coagulable lymph.—*Note of the Author.*

be conquered prior to effusion. The water-stroke is the actual original disease, and not, as hydrocephalus is, the accidental termination of some other affection.

The causes of water in the brain are predisposing, and occasional or exciting; and these latter may be divided into such as act primarily on the brain, and primarily upon the general system, and remotely on the brain; or, as it is sometimes termed, by sympathy. Fever, of whatsoever kind, is one of the most frequent causes of effusion, and this effusion is not uncommonly found after death. Scarlet fever, in which a very considerable degree of vascular excitement and disturbance prevails within the brain, is not an unfrequent cause; yet it is the reverse when considerable hectic fever or chronic debility remains after an attack of small-pox or measles. In proportion to the irritability of the system, which irritability is frequently produced, and is invariably kept up, by a state of *debility*, is its liability to be affected by the exciting causes of diseases. Fever, therefore, more commonly exists in children, and is easily occasioned by fatigue, dentition, worms, &c. Costiveness is a frequent occasional or exciting cause of hydrocephalus, and it may operate in two ways; *first*, by causing a greater determination of blood to the head; and, *secondly*, the fever, which results from retained fæces, may affect the brain in a greater degree than other parts; it being usual for proper fever to act on certain parts with more or less intensity, and thus to endanger effusion. All debilitating causes should be sedulously avoided, because generative of irritability, such as bleeding, especially in the exanthematous fevers, and other depletory methods, not overlooking the injudicious exhibition of mercurial medicines. The present plan of education, in which the intellectual powers are prematurely exercised, may be considered as one of the causes of the more frequent occurrence of hydrocephalus; and to this may be added, concussion or agitation of the brain; also blows on the head, although not very violent; yet Dr. Yeats's* explanation, as well as other authors', is extremely unsatisfactory of the mode in which they produce their effect. Some writers say that hydrocephalus frequently arises from *sympathy*, with diseases of the liver. This assertion is not corroborated by Golis, who never mentions any affection of this viscus, although he appears generally to have opened the abdomen, and reports, for the most part, a healthy condition of *all* the alvine viscera. Cheyne is a strenuous supporter of this hypothesis of sympathy; and Mr. Thompson, of Sloane-street, says he found inflammatory affec-

* Statement of Early Symptoms, &c. p. 24.

tion of the liver in nine out of eleven dissections. Dentition is a frequent cause of hydrocephalus, and it produces this effect in two ways; *first*, by acting as an exciting cause of fever, during which the increased vascular excitement preponderates in an inordinate degree in the cerebral organs; *secondly*, the local irritation in the gums is more immediately communicated to the brain by sympathy, and the consequences of this cerebral irritation are inordinate vascular action, and correspondent exhalation, especially in children who are constitutionally predisposed thereto, and in whom effusion sometimes arises during dentition, and prior to the manifestation of any febrile symptoms.

*General Principles of Treatment.* When the symptoms have been preceded by worms, or constipation, purgatives will rank among the most efficacious agents; and they are equally so whether these morbid states favour an undue determination of blood to the brain, or produce sympathetic irritation in the membranes of that organ, and consequent inflammation. It is here we are most successful. The kind of purgative is immaterial, provided the intestines be completely emptied, and the always vitiated state of the secretions restored to a natural and healthy condition. A combination of scammony, jalap, and calomel is highly useful;—and it is better to give a dose of calomel at night, and senna united with jalap on the following morning, than to exhibit calomel alone as a purgative, or to promote its action by Epsom salts, or other saline neutrals. But when fever exists, the treatment should be adapted to that fever; and its disposition to invade particular organs, must not be forgotten, so as to obviate the danger of structural disease. General blood-letting is objectionable; yet, the application of leeches is advisable, when there exist evident signs of increased determination to the brain. No class of medicines is better adapted to the combined state of fever and irritability than sedatives and diuretics; and considerable benefit has been derived from their administration in hydrocephalus. The mineral acids, joined with squill or digitalis, exert a beneficial effect in diminishing the force of the exacerbation, and controlling general and local inordinate vascular action. It is chiefly in counteracting the predisposition that we can hope to be eminently successful. The means of effecting this are to be sought for in those medicines which lessen irritability in the system generally, and moderate the inordinate circulation in the brain. As general irritability is found to exist, in a great degree, in children of a weakly habit, much service may be expected from tonic remedies, yet, they must not exert considerable stimulant power when the debility is conjoined with increased action of the vascular system. When simple debility exists alone, occasioned either by too long continuance at the breast, or by the use of improper diet, some of the preparations of iron are highly advantageous. Among these, the tartrate of iron is one of the most commodious, for, soluble in aqueous fluids, it can be exhibited with great facility. The *liquor ferri alkalini* of the London Pharmacopœia is also a useful form. When increased vascular action is present, the mineral acids are preferable to metallic tonics. In all of these cases the diet is to be nourishing and invigorating, such as is easily digestible, and producible of abundant chyle. Irregular and improper food is highly detrimental to the growth and health of children, and the quality of it is oftentimes more hurtful than the quantity. The analysis will be appropriately terminated with the concluding sentence of the author.

"My object is to direct the attention of practitioners to the view I have taken of acute hydrocephalus; that of not considering it as a proper idiopathic disease, but the effect of some previously existing disease, the most frequent of which is fever; or, as the consequence of increased exhalation, the natural result of simple increased vascular excitement, arising from various causes, acting in children of debilitated constitutions, or irritable habits; and to point out the obvious inutility of combating a single symptom, in place of embracing a comprehensive view of the essential character, usual progress, and natural termination of the preceding or existing disease.

Whether Dr. Shearman be right or wrong in his opinions, we have great pleasure in leaving the profession to determine. It is a subject, on which every reader can judge for himself, and he is enabled so to judge by the analysis that has been presented. We must, however, observe that the writer, who voluntarily attempts to convict the whole medical world of error, who accuses it of mistaking a mere morbid effect for an idiopathic disease, who claims for himself that peculiar perspicacity which Nature has denied to most other men, ought to be scrupulously cautious in the assertions and arguments which he employs, and most strictly philosophical in the deductions which he draws from them.—Again, writing, as his bounden duty is, for truth and not for victory, he should betray no disposition to sacrifice well-known facts to preconceived hypotheses. How far Dr. Shearman has done either the one or the other, will be ascertainable by the succeeding observations. He first tells his readers (p. 48,) that the character of the hydrocephalic fluid, demonstrates it not to be the product of inflammation, because it is not coagulable; and, secondly, he affirms, that in peritoneal and ovarian dropsies, resulting from membranous inflammation, the effused fluid is not only easily coagulable, but sometimes found actually coagulated. So far the distinction is complete and satisfactory. Yet, in the next sentence, he confesses that the hydrocephalic fluid is, at times, coagulable, and, although, he has just maintained that coagulation is the test of inflammatory action, and adduced this circumstance for that special purpose, he gravely asserts that, in this particular instance, coagulation is no proof of the existence of inflammation, but simply the result (p. 49) of strong vascular excitement, which, be it recollected, he judiciously declares (p. 39) to be different from inflammation. If then, according to his own admission, coagulation of the deposed fluid be capable of occurring in vascular excitement as well as in actual inflammation, why did Dr. Shearman display so miserable a specimen of logic as triumphantly to cite this fact to establish the truth of his darling hypothesis? Here he appears to have fallen into the error which logicians designate, *Ignoratio Elenchi*. But the author goes farther still; with him,

> " ———— no prodigies remain,
> " Comets are regular, and Wharton plain."

He informs his readers (p. 38) that, "if aqueous effusion depended upon inflammation, the quantity of fluid should be greater, in proportion to the intensity of the inflammation." Now we appeal to the profession, whether this assertion be not directly contrary to sound observation. No quantity, scarcely any effusion commonly originates from intense inflammation, which soon advances into either gangrene or suppuration, whilst it is inflammation of the lowest grade that yields copious effusion, and which effusion is a kind remedial effort of Nature. Has this writer forgotten that violent peritoneal inflammation produces excessive pain, and not unfrequently destroys life in fifty hours, whereas another inflammation of that identical membrane (his own opinion of the proximate cause of ascites, *Note*, p. 48) comes on insidiously, continues for months, is unattended with pain, and, finally, relieves itself by filling the peritoneal cavity with water, which is always coagulable, and occasionally coagulated?

With these exceptions, we think Dr. Shearman has written, as he usually does, in a candid and sensible manner, and that his book deserves to be read with attention and respect; especially, the preface to it, which claims our entire approbation.

A few concluding observations yet remain to be made, and which were necessarily deferred until now, since, previously, they would have been misplaced and pointless. We wish to call the attention of the reader to the extraordinary, and, as far as intellectual improvement is concerned, the melancholy difference of opinion between Dr. Yeats and Dr. Shearman with respect to the cause of water in the brain. We protest against all misconception, and, therefore, distinctly state that our object is neither to exalt nor depreciate either of these authors, neither to blame, nor even to reprove them for doing what they had an undoubted right to do, and which, doubtless, they have done conscientiously, but only, by commenting upon such difference of opinion, to express observations, and to draw conclusions that may, perhaps, be useful to medical literature in general, and to our readers in particular. That Dr. Yeats is a well-informed and judicious physician, that he has produced a beneficial work on hydrocephalus internus, which can be read with advantage, and trusted to in emergency, seem to be almost incontrovertible propositions:—Dr. Shearman, also, is a man not to be named without respect, whose abilities are respectable, and whose opportunities have not been inconsiderable, and thus, nearly equally balanced, as these gentlemen appear to be, in the scales of talent and respectability, it is an inevitable conclusion that, if Dr. Shearman be right in his opinions, Dr. Yeats is deplorably wrong, and has published a book which is replete with error and founded upon doctrines that are false, and, on the other hand, if we reverse this conclusion, it is undeniable that Dr. Shearman has deluded himself and misled his readers, as far as in his power laid, by the advancement of an hypothesis that is vain and erroneous. Such is the dilemma in which one or other of these physicians is plunged, self-evident, as it must be, that both of them cannot be right, although, within the sphere of probability, that both may be wrong. Dr. Yeats declares water in the brain to be a strictly idiopathic disease—Dr. Shearman asserts it to be a mere accidental effect of disease—Dr. Yeats maintains it arises from inflammation within the cranium, induced either directly or indirectly—Dr. Shearman affirms it has no connexion whatever with inflammation, but is the result of excitement, favoured by fever and predisposition. Even this antithesis, pointed as it is, falls short of the tenacity with which each of these writers supports his peculiar opinions. So certain is Dr. Yeats of water in the brain springing from inflammation that, after condemning the appellatives of the disease now in use, he proposes to designate it *phrenicula hydrencephalica*, because, and his language shall be transcribed for the sake of literal accuracy, phrenicula "is sufficiently expressive of that inflammatory condition of the brain, which is sometimes destructive both to its energy, and to life without effusion of fluid: and I add hydrencephalica as the distinctive appellation, descriptive of that collection of fluid in the ventricles which is the most common termination when the disease ends fatally."—Page viii. Thus will our readers observe, and the observation is important, that this inflammation of the brain is not a random thought, or a hasty suggestion of Dr. Yeats, but an absolute fact, about which he entertains not the slightest doubt, since he unhesitatingly recommends a new name for hydrocephalus internus, declarative of that inflammatory condition which he deems to be its constant and essential characteristic. Dr. Shearman confidently contends, that practitioners fall into error,—"calling by the name of hydrocephalus acutus, those diseases of the head which are actually and decidedly inflammatory; confounding together diseases which are in themselves essentially different, and inferring, without sufficient grounds, that when effusion is detected, there must have been inflammation; and that when inflammation of the brain or its membranes is present, the disease must be hydrocephalus."—Page 35.

Such is the astounding contrariety of sentiment, which obtains between two metropolitan physicians, not young, not ignorant, not inexperienced, living in the same age, and practising among the same population, and apparently candid and sensible individuals, who, may be supposed to have outlived the indiscretion of youth, and who, in the maturity and solidity of middle life, may be presumed to be exempt from that mutability of opinion and that temerity to declare it, which are the memorable foibles of juvenile and imbecile minds. With facts so convincing as these forcing themselves upon the public attention, who can believe the practice of medicine to be other than a mere conjectural art, or who ought to read the various medical productions of the day, without exercising no common degree of circumspection, scepticism, and judgment? That these, and similar facts are of frequent occurrence can scarcely be questioned, that the explanation of their origin is easy will not be doubted by those that understand human nature, and that they arise from causes which will never cease to operate, is an assertion that few will be disposed to controvert.* Since then, to read, is sometimes only to be misin-

* We are anxious to leave nothing unsaid on the important subject of water in the brain. Until now, we have designedly abstained from recording and commenting on a singularly unguarded sentence in the preliminary observations of Dr. Yeats, wherein he observes, "this disease will not unfrequently prove fatal under the best treatment from the beginning. From various causes, like any other disease, as peripneumony for example, than which hydrocephalus does not appear to be more dangerous."—P. xx. If this be not an unfounded and extravagant assertion, words have lost their meaning.

formed, and to seek for truth is not always to find it, and when, at best, errors are to be avoided, theories distrusted, and perplexities to be disentangled, the readers, who are anxious to gain the path of correctness, will act wisely in adopting the oracular advice of a man, whose genius reflects lustre on his country, and who, with that intuitive sagacity, for which he was so remarkable, appears almost to have penned it for the present occasion.—" Read not to contradict and confute; nor to believe and take for granted; nor to find talk and discourse: but to weigh and consider."—*Lord Bacon.*

Far and wide is the difference between hydrencephalus and peripneumony. Peripneumony is a disease not to be mistaken, evident to the feelings, palpable to the senses, destitute of all uncertainty:—Hydrencephalus is the antipous to these, is surrounded with mystery, and its very existence never undeniably determined but by dissection. Peripneumony is notoriously controllable by judicious and energetic agents, probably beyond every other phlogistic attack:—Hydrencephalus is decidedly the converse, it may or may not be that affection, for it is simulated by many morbid conditions, and hence, in every instance, the cure rests on a mere gratuitous assumption of the reality of the disease. All which Dr. Yeats can conceivably mean, is, that the premonitory symptoms may be so cured, yet, where is his proof of these symptoms, even if disregarded, advancing into hydrencephalus, since there is nothing certainly diagnostic in them?—Again, Dr. Yeats has strangely forgotten himself, for in the same breath with which he uttered the assertion dwelt upon, he confessed that hydrencephalus " will not unfrequently prove fatal under the best treatment." Where, then, is the comparison which he instituted? Will he affirm, in defiance of the experience of the majority of the profession, that peripneumony " will not unfrequently prove fatal under the best treatment?" And if he will not do so, is not the conclusion obvious?—*Rev.*

